# Editorial: Immunological Mechanisms, Biomarkers and Immunotherapies of Alzheimer's Disease

**DOI:** 10.3389/fnagi.2021.733282

**Published:** 2021-09-13

**Authors:** Jia-yan Xin, Xiao-yan Zhu, Xiao Huang, Yu-hui Liu, Jun Tan, Yang Xiang

**Affiliations:** ^1^Department of Clinical Medicine, North Sichuan Medical College, Nanchong, China; ^2^Department of Neurology, General Hospital of Western Theater Command, Chengdu, China; ^3^Basic Medical Laboratory, General Hospital of Western Theater Command, Chengdu, China; ^4^Department of Neurology and Centre for Clinical Neuroscience, Daping Hospital, Third Military Medical University, Chongqing, China; ^5^Department of Obstetrics and Gynecology, Affiliated Hospital of Guizhou Medical University, Guiyang, China; ^6^Department of Neurology, Sichuan Provincial People's Hospital, University of Electronic Science and Technology of China, Chengdu, China; ^7^Chinese Academy of Sciences Sichuan Translational Medicine Research Hospital, Chengdu, China

**Keywords:** immunological mechanism, biomarker, immunotherapy, Alzheimer's disease, peripheral system

Alzheimer's disease is the most common type of dementia characterized by neuropathological changes such as intracellular tau tangles and extracellular deposition of β-amyloid (Aβ) as plaques. At present, the study of pathogenesis, the search for biomarkers, and the development of treatment strategies of AD are all closely related to immunological theory and techniques. The main aim of the current Research Topic was to provide a current collection of immunological mechanisms, biomarkers, and therapeutic strategies in AD.

Growing evidence has proved that Alzheimer's disease (AD), as a typical degenerative disease of the central nervous system, has complex connections with the peripheral system (Wang et al., 2017), such as the peripheral clearance of pathogenic substances (Aβ and Tau) or the impact of gut microbiota on AD-like pathological characteristics, etc. Among them, the relationship between systemic autoimmune diseases and AD has been puzzling researchers for years. Culibrk and Hahn summarized the link of chronic inflammatory osteoarthritis disease with late-onset AD. In particular, they focused on the pathophysiological characteristics and immunotherapy of rheumatoid arthritis, osteoarthritis, and osteoporosis, as well as their implications on AD pathogenesis. They also summarized some other possible mechanisms, including the age-related cellular/immuno-senescence, the aberrant peripheral nervous system activity, dysregulated autophagic homeostasis, and the pathological microRNA profiles, which could attract more attention from the audience.

The relation between hereditary amyloidosis and AD is a topic rarely discussed before. Although the pathogenic substances of the two diseases are different, it is not an obstacle to investigating their association from the perspective of genetics. Jiang et al. reported two novel likely pathogenic frame-shift mutations of gelsolin in AD patients using the genes targeted sequencing (GTS) method. Intriguingly, both mutations seem to correlate with the age at which clinical symptoms of AD appeared, respectively, even though the gelsolin is a common pathogenic gene charged with hereditary amyloidosis.

The development of biomarkers is a spotlight in AD research over the past decade. The proposal of the NIA-AA research framework of AD in 2018 not only categorizes AD biomarkers as per the AT(N) system but also provides more objective and rigid biological criteria for the diagnosis of AD than ever before (Jack et al., 2018). Since the presence or absence of Aβ is the sole criterium for the etiologic diagnosis of AD in the AT(N) system, the emerging novel non-Aβ biomarkers are mostly classified in the (N) catalog. Based on the Alzheimer's Disease Neuroimaging Initiative (ADNI) cohort, Pillai, Bebek et al. measured the levels of tumor necrosis factor receptor (TNFR) 2 in the cerebrospinal fluid (CSF) and investigated its correlations with CSF t-Tau, CSF p-Tau, the cognitive domains, the MRI measures, and the longitudinal cognitive changes, together indicating the potential value of TNFR2 in the diagnosis and treatment of AD.

The biomarker research has gradually shifted from CSF to blood due to the invasive operation, difficult acquisition, and patient unacceptability of CSF sampling (Zetterberg and Blennow, 2018). Wang J. et al. compared the naturally occurring antibody to α-synuclein (NAb-a-Syn) in the blood of patients with Parkinson's disease dementia, AD, vascular dementia, and normal controls, and further explored the correlations between NAb-a-Syn and the severity of cognitive impairment and PD, respectively. Of note, the naturally occurring antibodies also have a good performance in the immunotherapy of AD (Liu et al., 2021).

As a shining star of AD biomarkers in the past years, the value of neurofilament light chain (NfL) in AD diagnosis and prognosis is no longer needed to be described. Especially, the blood-NfL is consistent and comparable with CSF-NfL, which greatly increases the clinical application value of NfL (Fortea et al., 2018). Wang J-H. et al. evaluated the difference of blood-NfL in patients with post-stroke subjective cognitive decline (SCD). Although this study was a single-center prospective study with a small sample size, post-stroke SCD is a clinical condition that has received little attention before, showing the careful observation and thinking of the authors.

Likewise, neurogranin has emerged as an important biomarker for neurodegenerative diseases in recent years, although the current studies have not demonstrated that its clinical value is comparable to that of the NfL. Xiang et al. reviewed the associations between neurogranin and neurological and psychiatric diseases and further made the perspectives on the research and applications of neurogranin in the future.

Compared with body fluid-based biomarkers, image-based biomarkers have not developed rapidly over recent decades. The clinical applications of Aβ-positron emission tomography (PET) and tau-PET have promoted the early diagnosis and severity assessment of AD (Hansson, 2021); however, the high cost and low coverage of PET limit its clinical application. Many reports on functional magnetic resonance imaging (fMRI) emerged, but fMRI has not yet reached the same value as that of Aβ-PET and tau-PET in the diagnosis of AD. Based on ADNI, Yang et al. studied the dynamics and concordance changes in different brain regions in the patients with SCD using resting-state fMRI (RS-fMRI), adding the new evidence for the use of fMRI in AD.

Researchers have long been interested in acupuncture for the treatment of neurological diseases, even though its mechanism is not fully understood (Kaptchuk, 2002). Based on growing published data, Huang et al. analyzed the therapeutic effect of acupuncture on AD using a meta-analysis method. The authors proposed that high-quality studies with rigorous study designs and larger samples are required in the future, even though it is now generally believed that acupuncture is a promising complementary treatment for AD.

Over the past decades, a variety of hypotheses about the mechanism of AD have emerged. At present, even the amyloid cascade hypothesis either have to face updates or have to face challenges (Tolar et al., 2020). A growing body of evidence suggests that the mechanisms of AD beyond Aβ and Tau are possible in which the neurovascular unit, brain lymphatic system, gut microbiota, and neuroinflammation could be involved (Henstridge et al., 2019). Among them, the activation of the immune system is the key regulatory factor of AD pathology (Long and Holtzman, 2019). Moreover, almost all the clinical trials of anti-AD drugs targeting Aβ or Tau failed or have been inconclusive (Mullard, 2021), suggesting that the immunotherapy strategies may also need to concentrate on some other targets rather than just Aβ and Tau.

We have always regarded AD as a degenerative disease of the central nervous system. However, more and more evidence confirms that the peripheral system has a great influence on AD. As an essential part of the peripheral system, the role played by the immune system and tissues and organs rich in immune cells in the pathogenesis of AD have not been fully answered. The spleen, intestine, omentum, and blood, these seemingly distant and unrelated organs and tissues are likely to affect our brain structure and cognitive function *via* immunological regulation, fascinating scientific issues for further and future research.

## Author Contributions

All authors listed have made a substantial, direct and intellectual contribution to the work, and approved it for publication.

## Funding

This study was supported by the National Natural Science Foundation of China (81601112, 81801090), Top Project of the Youth Incubation Program of Military Medical Science and Technology (19QNP065), and the Sichuan Department of Science and Technology Fund (2018SZ0141, 2019YSF0213).

## Conflict of Interest

The authors declare that the research was conducted in the absence of any commercial or financial relationships that could be construed as a potential conflict of interest.

## Publisher's Note

All claims expressed in this article are solely those of the authors and do not necessarily represent those of their affiliated organizations, or those of the publisher, the editors and the reviewers. Any product that may be evaluated in this article, or claim that may be made by its manufacturer, is not guaranteed or endorsed by the publisher.
